# Characterisation of a putative M23-domain containing protein in *Mycobacterium tuberculosis*

**DOI:** 10.1371/journal.pone.0259181

**Published:** 2021-11-16

**Authors:** Andrea Olga Papadopoulos, Christopher Ealand, Bhavna Gowan Gordhan, Michael VanNieuwenhze, Bavesh Davandra Kana

**Affiliations:** 1 Faculty of Health Sciences, DSI/NRF Centre of Excellence for Biomedical TB Research, School of Pathology, University of the Witwatersrand, National Health Laboratory Service, Johannesburg, South Africa; 2 Department of Chemistry, Indiana University Bloomington, Bloomington, Indiana, United States of America; Bose Institute, INDIA

## Abstract

*Mycobacterium tuberculosis*, the causative agent of tuberculosis remains a global health concern, further compounded by the high rates of HIV-TB co-infection and emergence of multi- and extensive drug resistant TB, all of which have hampered efforts to eradicate this disease. As a result, novel anti-tubercular interventions are urgently required, with the peptidoglycan component of the *M*. *tuberculosis* cell wall emerging as an attractive drug target. Peptidoglycan M23 endopeptidases can function as active cell wall hydrolases or degenerate activators of hydrolases in a variety of bacteria, contributing to important processes such as bacterial growth, division and virulence. Herein, we investigate the function of the Rv0950-encoded putative M23 endopeptidase in *M*. *tuberculosis*. *In silico* analysis revealed that this protein is conserved in mycobacteria, with a zinc-binding catalytic site predictive of hydrolytic activity. Transcript analysis indicated that expression of Rv0950c was elevated during lag and log phases of growth and reduced in stationary phase. Deletion of Rv0950c yielded no defects in growth, colony morphology, antibiotic susceptibility or intracellular survival but caused a reduction in cell length. Staining with a monopeptide-derived fluorescent D-amino acid, which spatially reports on sites of active PG biosynthesis or repair, revealed an overall reduction in uptake of the probe in ΔRv0950c. When stained with a dipeptide probe in the presence of cell wall damaging agents, the ΔRv0950c mutant displayed reduced sidewall labelling. As bacterial peptidoglycan metabolism is important for survival and pathogenesis, the role of Rv0950c and other putative M23 endopeptidases in *M*. *tuberculosis* should be explored further.

## Background

Tuberculosis (TB), caused by *Mycobacterium tuberculosis* infection, has surpassed HIV/AIDS as the leading cause of death by an infectious disease globally [1]. Currently, rates of decline in TB incidence are insufficient to reach the ambitious targets set by the World Health Organization in the End TB Strategy including reduction of TB deaths and new infections by 95% and 90% respectively by 2035. To achieve these goals, novel transmission blocking and health system strengthening strategies are needed, together with the development of a shorter, more effective treatment regimen [1]. In this context, the mycobacterial cell wall is attractive for identification of new drug targets as most components and biosynthetic enzymes required to create and maintain this structure are not present in eukaryotes. The cell envelope in mycobacteria consists of a multi-layered, highly complex structure made up of a peptidoglycan (PG) core linked to arabinogalactan and mycolic acid layers [2]. PG is made up of repeating disaccharides consisting of *N*-acetyl glucosamine (Glc*N*ac) and either *N*-acetyl muramic acid (Mur*N*ac) or *N*-glycolyl muramic acid (Mur*N*Glyc), which is further covalently linked to a stem-peptide comprising L-Alanine, D-Glutamate, *meso* diaminopimelic acid (mDAP) and a terminal D-Alanine dipeptide [3,4]. Bacterial PG is typically stabilised by cross-links between the 4^th^ D-Alanine and 3^rd^ *m*DAP (4–3 cross-links) of the stem-peptide but mycobacterial PG has a characteristically high proportion of 3–3 cross-linking, particularly abundant during stationary phase [4]. Cross-bridges, consisting of glycine residues have also been identified between *m*DAP residues of mycobacteria [4].

Successful growth, proliferation and persistence of mycobacterial cells relies on the synergistic synthesis and hydrolysis of the PG core by a variety of enzymes that act on different substrates. Hydrolases, in particular have important roles in cell division and if dysregulated, can lead to autolysis [5–8]. *N*-acetylmuramoyl-L-alanine amidases hydrolyse the amide bond between the L-Alanine of the stem peptide and muramic acid of the sugar backbone to degrade PG. In *Mycobacterium smegmatis*, the *ami1*-encoded amidase is required for septal PG hydrolysis to allow for daughter cell separation and also plays a role in fatty acid synthesis, while the *M*. *tuberculosis* homologue, Rv3717, acts in combination with the RipA hydrolase for cell division [9–11]. Another amidase in *M*. *smegmatis*, CwlM (or Ami2), is essential for regulating the first steps of PG biosynthesis. The *M*. *tuberculosis* CwlM homologue displays phospho-regulatory roles and interacts with FhaA, a protein required for cell elongation [12–14]. Given the central role of some of these hydrolases in the cell cycle, it follows that their activity is carefully regulated to prevent untimely degradation of the cell wall and cell lysis.

The *Escherichia coli* genome encodes numerous amidase homologues that degrade septal PG to facilitate daughter cell separation. This process is regulated through the N-terminal AMIN domain, which specifically regulates septal localisation and PG binding [15–20]. The C-terminal amidase-3 domain has a catalytic site occluded by an auto-inhibitory α-helix and as a result, these amidases can only degrade PG in the presence of the M23 endopeptidase activators EnvC or NlpD [16,21]. Consequently, the loss of *envC* causes filamentation in *E*. *coli* and conditional lethality upon depletion of MinCDE division regulation system [22]. Collective loss of *nlpD* and *envC* further exacerbated the cell division defect in *E*. *coli*, which was worsened by depletion of two additional M23 endopeptidases, YebA and YgeR [23]. YebA, shown to degrade intact PG and convert cross-linked tetrapeptide residues into monomeric tetrapeptides, is jointly essential with two NlpC/p60 proteins for *E*. *coli* growth [24].

The M23 endopeptidase domain is characterised by antiparallel β-sheets with a zinc-coordinating active site that can hydrolyse PG. However, there are M23 endopeptidases that have degenerate M23 domains, such as EnvC and NlpD from *E*. *coli*, with no catalytic activity per se but instead, interact with amidases to regulate their activity. While numerous classes of PG remodelling enzymes have been identified in mycobacteria, the M23 endopeptidases, either as degenerate amidase activators or as active peptidases, have not been studied in *M*. *tuberculosis*. Herein, we set out to identify putative M23-endopeptidase encoding genes in *M*. *tuberculosis*. Of the three M23 domain-containing genes identified, Rv0950c was found to carry a conserved zinc-binding catalytic site and deletion thereof resulted in cell shortening and reduced incorporation of fluorescent D amino acids (FDAAs).

## Materials and methods

### Phylogeny and homology modelling of bacterial PG remodelling enzymes

Protein sequences acquired from NCBI and UniProt (http://www.uniprot.org/uniprot/) [25] were used to generate a multiple sequence alignment of characterised M23 endopeptidases, NlpC/p60 domain proteins, L,D transpeptidases (Ltds) and DD-carboxypeptidases in Clustal Omega (http://www.ebi.ac.uk/Tools/msa/clustalo) [26]. A phylogenetic tree was subsequently generated in Trex-online (http://www.trex.uqam.ca/) [27]. pFAM-predicted domain architecture was added as annotations [28]. To confirm the presence of a putative M23 endopeptidase domain in Rv0950c, homology modelling was performed based on the highest sequence homology between Rv0950c and previously crystallised proteins uploaded to the Swiss Protein Database [29]. The model was viewed and analysed using the Swiss PDB viewer. The amino-acid sequence of Rv0950c obtained from Mycobrowser (https://mycobrowser.epfl.ch) was used as the template sequence. A graphic model for Rv0950c was generated in Swiss PDB viewer.

### Characterisation of the zinc-binding motifs in the putative *M*. *tuberculosis* M23 endopeptidases

In order to determine which residues align with the zinc-coordinating HxGxD and HLH motifs of *S*. *aureus* LytM, a multiple amino-acid sequence alignment was performed between the putative M23 endopeptidase domains of the *M*. *tuberculosis* homologues and various characterised M23 endopeptidases, using Clustal Omega. Amino-acid sequences of non-mycobacterial M23 endopeptidase domain sequences were obtained from NCBI. Mycobrowser [30] and the PHMMER database [31] were used to identify homologues of the putative *M*. *tuberculosis* M23 endopeptidases in other mycobacterial species.

### *Mycobacterium tuberculosis* growth

Strains of *M*. *tuberculosis* (S1 Table) were grown in vented, sealed-cap culture flasks or 9 cm petri dishes containing either Middlebrook 7H9 broth supplemented with oleic acid-albumin-dextrose-catalase (OADC) (10%), glycerol (0.2%) and Tween-80 (0.2%) or Middlebrook 7H11 solid media supplemented with OADC (10%), and glycerol (0.5%). Middlebrook 7H9 and 7H11 were further supplemented with antibiotics [Kanamycin Sulphate (25 μg/ml) and/or Hygromycin B (50 μg/ml)] where applicable (S1 Table). Cultures were grown at 37°C. For growth curves, cultures of *M*. *tuberculosis* were sub-cultured to OD_600nm_ = 0.02 in 7H9 (50 ml) and growth was monitored by OD_600nm_, with a portable WPA Cell Density Meter and corresponding CFU/ml was determined by plating a dilution series at days 2, 4, 7, 9, 11, 14, 16 and 18.

### Growth phase-dependent transcription analysis

Transcription profiles of putative M23 endopeptidase genes of *M*. *tuberculosis* were studied in relation to growth phase. At OD_600nm_ = 0.4, 0.8, 1.2, and 2.0, 10 ml of cells were harvested by centrifuging at 4000 rpm, for 10 min at 4°C. The cell pellets were resuspended in 1 ml Trizol reagent (Sigma) spiked with poly-Aryl carrier (10 μl), transferred to lysing matrix B tubes (MP Bio) and disrupted three times for 1 min intervals (using a RiboLyser, Roche) with incubation on ice for two min in between. The lysates were stored at -80°C until harvesting of each growth phase sample was complete. RNA was extracted by Trizol-phenol chloroform and converted to cDNA using gene specific reverse primers (S2 Table) and the Superscript III RT kit (Invitrogen). cDNA was quantified using SybrGreen-based qPCR alongside a 10-fold genome equivalent standard dilution using the SsoFast Sybrgreen chemistry and BioRad CFX96 Real-Time thermocycler. The calculated concentration (Sq) values for each reaction, based on genomic DNA standard curves, were copied into Excel for further analysis. To normalise the calculated expression of the gene of interest (GOI) to that of the reference gene (*sigA*), the following formula was used: Sq of GOI/Sq of *sigA*.

### Gene deletion via suicide vector-mediated homologous recombination

Vectors used for genetic manipulations are listed in S3 Table. An unmarked deletion of Rv0950c was performed via suicide vector-mediated two-step allelic exchange [32]. Mycobrowser [30] was used to acquire the sequence of Rv0950c with additional upstream and downstream sequences. Clone Manager 9.0 was used to select upstream and downstream homologous regions which could be fused together for in-frame deletion of the ORF, without disrupting potential neighbouring genes and/or their putative promotor regions. Primers were designed in Primer3 (http://primer3.ut.ee/) and used for amplification of the upstream and downstream arms with flanking restriction sites and GC-repeat hexamers for cloning into the MCS of p2Nil (S4 Table). The upstream region was first cloned into pBlueScript [33] to yield pBlue09US (S3 Table). Thereafter, the upstream region was excised from this vector and was cloned together with the downstream region (amplified by PCR and subsequently digested with restriction enzymes) into p2Nil to yield p2NilΔRv0950c. The *lac_sac* cassette from pGOAL17 was then cloned into the *Pac*I sites of this plasmid to complete construction of the knockout vector p2NilΔ09SV (S3 Table) [32]. The resulting in-frame deletion prevented disruption of neighbouring genes *uvrD1* and *succC*. The downstream homology arm in particular overlaps a putative promotor region of *succC*, which was retained in the deletion allele, whilst excluding the entire M23 endopeptidase domain.

Suicide vectors were extracted from *E*. *coli* using the Nucleobond Kit (MN) and electroporated into *M*. *tuberculosis* H37Rv(s) using a 2 mm GenePulser Cuvette (BioRad) and pulsed using the GenePulser XCell system (BioRad) at 2500 V, 25 μF, 1000 Ω. After rescuing in 7H9 media at 37°C overnight, the cells were plated on 7H11 supplemented with Kanamycin (25 μg/ml) and 0.004% X-Gal and then incubated at 37°C. The emergence of colonies was monitored over a period of 2–6 weeks. Blue, Kanamycin-resistant *M*. *tuberculosis* colonies represented potential single-crossover clones, which were then inoculated into 7H9 supplemented with Kanamycin (25 μg/ml) and the genotype confirmed using primers shown in S5 Table. These transformants were sub-cultured in 7H9 without kanamycin to induce the second crossover event and subjected to a 10-fold dilution series, which was then plated on 7H11 supplemented with 0.004% X-Gal and 2% sucrose to select for white, sucrose-resistant colonies which represented potential deletion mutants. These white sucrose resistant colonies are expected to have undergone a second cross-over event to either regenerate the wild type locus or to incorporate the mutant allele. During this process, the vector (which confers kanamycin resistance) is excised out of the genome and the resulting mutant is kanamycin sensitive. To further confirm loss of the vector, the mutant strain was replica plated on media with and without kanamycin (S1 Fig). The resulting mutant was genotyped by PCR (S1 Fig) and by Southern blot analysis and real-time PCR to confirm loss of Rv0950c gene expression (S2 Fig).

For PCR genotyping of strains, Faststart Taq polymerase (Roche *Biosciences*) was used to genotype *M*. *tuberculosis* H37Rv, ΔRv0950c and the single cross-over [SCO] strain used to create ΔRv0950c, using the genotyping primers given in S5 Table. PCR reactions were set up in 50 μl volumes containing Faststart PCR buffer (1x), GC enhancer (1X) (Roche), MgCl_2_ (2 mM), dNTPs (200 μM), gene-specific forward and reverse primers (0.4 μM) and Taq polymerase (1U). Two microliters of genomic DNA was used as template in the reaction whereas a reaction containing no DNA was used as a control. The thermocycling program (Bio-Rad, T100 thermal cycler) used was as follows: 95°C for 5 min; 40x cycles (95°C for 30 s, 64°C for 40 s, 72°C for 45 s); 72°C for 10 min. PCR amplicon sizes were then determined by running 20 μl of each reaction on a 2% TAE agarose gel (95 V for 60 min) for comparison of expected sizes.

### Genetic complementation

The complementation cassette consisted of the Rv0950c gene and additional upstream sequence of 300 bp representing the putative native promotor. The complementation cassette was amplified by PCR with restriction sites (S4 Table) for directional, sticky-end cloning into pTweety [34] to yield pT09 (S3 Table). The resulting vector was electroporated into the ΔRv0950c mutant and the transformants confirmed by PCR and Southern blot analysis. Genetic complementation of Rv0950c was confirmed by measuring restoration of gene expression by RT-qPCR (S2 Fig).

### Assessment of colony morphology

Mycobacterial strains (RvS, ΔRv0950c and ΔRv0950c::Rv0950c) were grown to mid-exponential phase (OD_600nm_ ~ 0.5) in 7H9 media (Middlebrook, Sigma) supplemented with OADC and 0.05% Tween80. Ten microliters of each strain was aliquoted onto solid 7H11 media (Middlebrook, Sigma) and allowed to dry completely. Plates were then incubated for 21 days at 37°C. Images of the entire colony were captured using a digital camera (Nikon) and higher magnifications of the colony center or edge were captured using a stereo microscope (Zeiss Stemi 2000 microscope running Zen 2011, blue edition software).

### Determination of MICs

For susceptibility assessments with antibiotics and lysozyme, axenic pre-cultures were grown and sub-cultured to OD_600nm_ = 0.1. The sub-cultures were grown overnight to OD_600nm_ = 0.2–0.4. Cultures were diluted 1:20 in 7H9 media. Thereafter, two-fold dilution series was setup in a clear, 96-well, round-bottom plate as follows: 7H9 (50 μl) was aliquoted into each well, except for the top row of 8 wells. Antibiotics or lysozyme was added to the top row at 128X the expected MIC in a final volume of 100 μl. Antibiotics were then diluted two-fold from the top row (row 12) to the bottom row (row 1), starting with transferring 50 μl from row 12 to row 11 containing 50 μl 7H9. For the bottom row, 50 μl was discarded, so that all wells contained a final volume of 50 μl. After dilution of the antibiotics and controls (media only or vehicle only), 50 μl of cells were added to each well, bringing the final volume up to 100 μl. Plates were sealed and incubated at 37°C and scored visually after 4–7 days, as growth or no growth in a well. The MIC was recorded for the lowest concentration of drug at which no growth was observed.

For menadione, mycobacterial strains were grown to mid-exponential phase before adjusting to an OD_600nm_ ~ 0.025. Cell suspensions were used to inoculate 96-well round-bottom plates that contained two -fold dilutions of antibiotics, lysozyme or a range of menadione concentrations (250–0.25 μg/ml). After incubating plates at 37°C for 5 days, 15 μl of alamarBlue^TM^ cell viability reagent (Invitrogen) was added to each well and further incubated at 37°C overnight (≥ 16 hours). Cell viability, and subsequent determination of MIC, was measured by assessing the fluorescence of reduced alamarBlue in a plate reader (excitation and emission at 530–560 and 590 nm, respectively). Three independent biological repeats were assessed.

### Ethidium bromide permeability assay

An axenic culture (20 ml) was grown to OD_600nm_ = 0.8, cells harvested, and washed in PBS (1 ml), and resuspended in 1X PBS and 0.5% glucose. Cells were added to a final OD_600nm_ = 0.4 (50 μl) in black, flat, clear-bottom, 96-well microplates (Greiner Bio). Ethidium bromide was added to a final concentration of 0.25 μg/ml to cells and made up to a final volume of 100 μl with nuclease-free water. Ethidium bromide fluorescence emission was measured with a Spectra Max Gemini EM microplate reader (Molecular Devices) at 1 min intervals for 60 min. Data was obtained from duplicate readings of three biological replicates for each strain and normalised to time zero readings. Statistical analysis was performed in Excel by one-way ANOVA.

### Scanning electron microscopy

Pre-cultures of *M*. *tuberculosis* were sub-cultured and grown to OD_600nm_ = 0.6–0.8 to capture cells at exponential growth phase, washed in 1X PBS and fixed overnight in 2.5% glutaraldehyde at 4°C. Cells were then washed in 1X PBS and resuspended in 2% osmium tetroxide for 60 min at room temperature, followed by dehydration in a range of ethanol concentrations (30, 50, 70 and 100%). Cell pellets were stored at 4°C before transfer to the Electron Microscopy Imaging Unit at the University of Cape Town for imaging where samples were prepared by spotting onto a filter followed by two Carbon coatings. Images were captured at 20000X magnification using the Tescan MIRA3 RISE SEM.

### Tetramethylrhodamine-3-amino-d-alanine (TADA) fluorescence microscopy

Axenic cultures of each strain were grown to OD_600nm_ = 0.6–0.7 and TADA (2.5 μl) was added to a 500 μl aliquot, followed by incubation at 37°C, for 30 min. Cells were harvested and washed in 1X PBS (Lonza) twice before fixing overnight in 250 μl of 2.5% glutaraldehyde (Sigma) at room temperature. Fixed cells were centrifuged at 13 000 rpm for 2 min and resuspended in PBS before spotting onto 1% agarose on glass slides, covered with a cover slip and sealed. Images were captured using the Nikon Eclipse T12 and PE4000 LED light source, in the TL DIC channel (200 msec exposure) and pre-configured TADA channel (excitation 550 nm, 50% power; emission 598 nm; 2 s exposure).

### Copper-catalysed azide alkyne cycloaddition (CuACC) detection of alkyne-DADA and fluorescence microscopy

Axenic cultures (5 ml) of each strain were grown to OD_600nm_ = 0.6–0.7. Cells were harvested and resuspended in 7H9 (100 μl) and labelled with alk-DADA (2.5 μl) at 37°C for 60 min. Cells were centrifuged, washed in 1X PBS (Lonza) and fixed overnight in 250 μl of 2.5% glutaraldehyde (Sigma) at room temperature. Fixed cells were centrifuged at 13 000 rpm for 2 min and resuspended in PBS-Tween-BSA (PBSTB) buffer (250 μl) and centrifuged at 13 000 rpm for 2 min. Pellets were resuspended in PBSTB (230 μl), to which CuACC reaction components were added in the following order: 10 mM CuSO_4_ (5 μl), 6.4 mM TBTA (5 μl), 60 mM Sodium Ascorbate (5 μl), 1 mM azido-probe (5 μl). The CuACC reaction was incubated at room temperature for 30 min and harvested by centrifuging the cells at 13 000 rpm for 2 min. The cells were washed three times in 1X PBS and added to an agarose pad as described previously. Images were captured using the Nikon Eclipse T12 and PE4000 LED light source, in the TL DIC channel (200 msec exposure) and pre-configured azo488 channel (excitation 490 nm, 50% power; emission 512 nm; 3 sec exposure).

### Lysozyme-Mutanolysin digestion prior to alkyne-DADA fluorescence microscopy

Axenic cultures (5 ml) of each strain were grown to OD_600nm_ = 0.6–0.7, harvested and resuspended in 7H9 (188 μl). Lysozyme (Roche) and Mutanolysin (Sigma) were added in 10-fold increasing concentrations (5–500 μg/ml lysozyme and 0.5 – 50U Mutanolysin) to a final volume of 250 μl, incubated at 37°C for 120 min. Cells were centrifuged and resuspended in 7H9 (100 μl) and labelled with alk-DADA (2.5 μl) at 37°C for 60 min. Cells were centrifuged, washed in 1X PBS (Lonza) and fixed overnight in 2.5% glutaraldehyde (250 μl) at room temperature. Fluorescence conjugation for incorporated alk-DADA was performed by CuACC as previously described.

### Fluorescence intensity analyses

Images were exported from Nikon Elements Software (unless otherwise stated) to.tif files to preserve original optical data and analysed in Fiji (ImageJ). The segmented line tool was used to trace the length of the cell from the brightest pole to the dimmest pole, for 100 cells for each biological replicate of each strain. The intensity analysis function (ctrl+k) was applied to acquire plot co-ordinates of fluorescence intensity (grey value) across the drawn line (y) with respect to pixel length of the line (x). The x and y coordinates were copied into Excel in categories of different staining patterns, eg. polar, mid-cell or diffuse, based on the shape of the intensity profile. Pixel length was normalised to a % of cell length and an average fluorescence intensity of the cells was calculated. The final fluorescence profile was derived from averaging the data points at 10% cell length intervals of up to ten cells with the highest grey values (brightest cells) for each replicate of each strain either the polar or mid-cell-stained cells. The spatial distribution of fluorescence between the bright pole (bp), side-wall (sw) and dim pole (dp) was calculated based on data published by García-Heredia et al., (2018), as follows:

fluorescenceofbp=∑greyvaluesfrom0to15%ofthecelllength

fluorescenceofsw=∑greyvaluesfrom16to85%ofthecelllength

fluorescenceofdp=∑greyvaluesfrom86to100%ofthecelllength

### Measurement of cell length in SEM micrographs

Images (.tif) were analysed using Fiji (ImageJ) using the pixel length tool. Pixel length was converted to μm in Excel by dividing cell pixel length by the pixel length of 1 μm. The pixel length of 1 μm was determined by dividing the 2 μm scale bar pixel length by two. For accuracy, all cells in one image were normalised to the scale bar of that image. Statistical significance of cell length was compared between strains by single-factor ANOVA of cell length measured for 100 separate cells in each of the three biological replicates (n = 300), at a confidence interval of 95%.

#### Determination of intracellular survival using activated THP-1 macrophages

THP-1 monocytes (Sigma) were grown and maintained in RPMI-1640 media supplemented with 10% FBS at 37°C, 5% CO_2_ for 7–10 days after which viability was assessed by trypan blue exclusion staining. When the density reached 2 x 10^5^ cells/ml, 1.5 ml of cells was seeded in each well of a 48-well plate. THP-1 monocytes were differentiated into adherent, activated macrophages by the addition of 100 nM phorbol myristate acetate (PMA) (Sigma), 150 U of recombinant human gamma interferon (IFN-γ) and incubation for 3 days at 37°C, 5% CO_2_. Cultures of RvS, ΔRv0950c and ΔRv0950c::Rv0950c were grown to exponential phase (OD_600nm_ 0.4–0.6) before adjusting to ~2 x 10^6^ cells/ml (OD_600nm_ = 0.05) in RPMI-1640 media supplemented with 10% FBS. Media in the wells containing adherent macrophages were then replaced with 1 ml of mycobacterial inoculum (10 bacteria: 1 THP1 cell) and incubated for a further 4 hours at 37°C, 5% CO_2_ to promote phagocytosis. Each well was then washed once and replaced with 1 ml, warmed RPMI-1640 media supplemented with 10% FBS. Ten microliters of 10% SDS was immediately added to one row of wells to lyse the macrophages which served as a Time zero. At appropriate time-points (Day 1, 3 and 5), mycobacterial cells were similarly collected for CFU/ml determination on 7H11 solid media (Middlebrook) which were incubated at 37°C for 4 weeks. Wells with uninfected macrophages served as mycobacteria-free controls at each time-point. Three biological replicate experiments were conducted.

## Results

### *M*. *tuberculosis* encodes three putative M23-like endopeptidases

**Fig 1:**
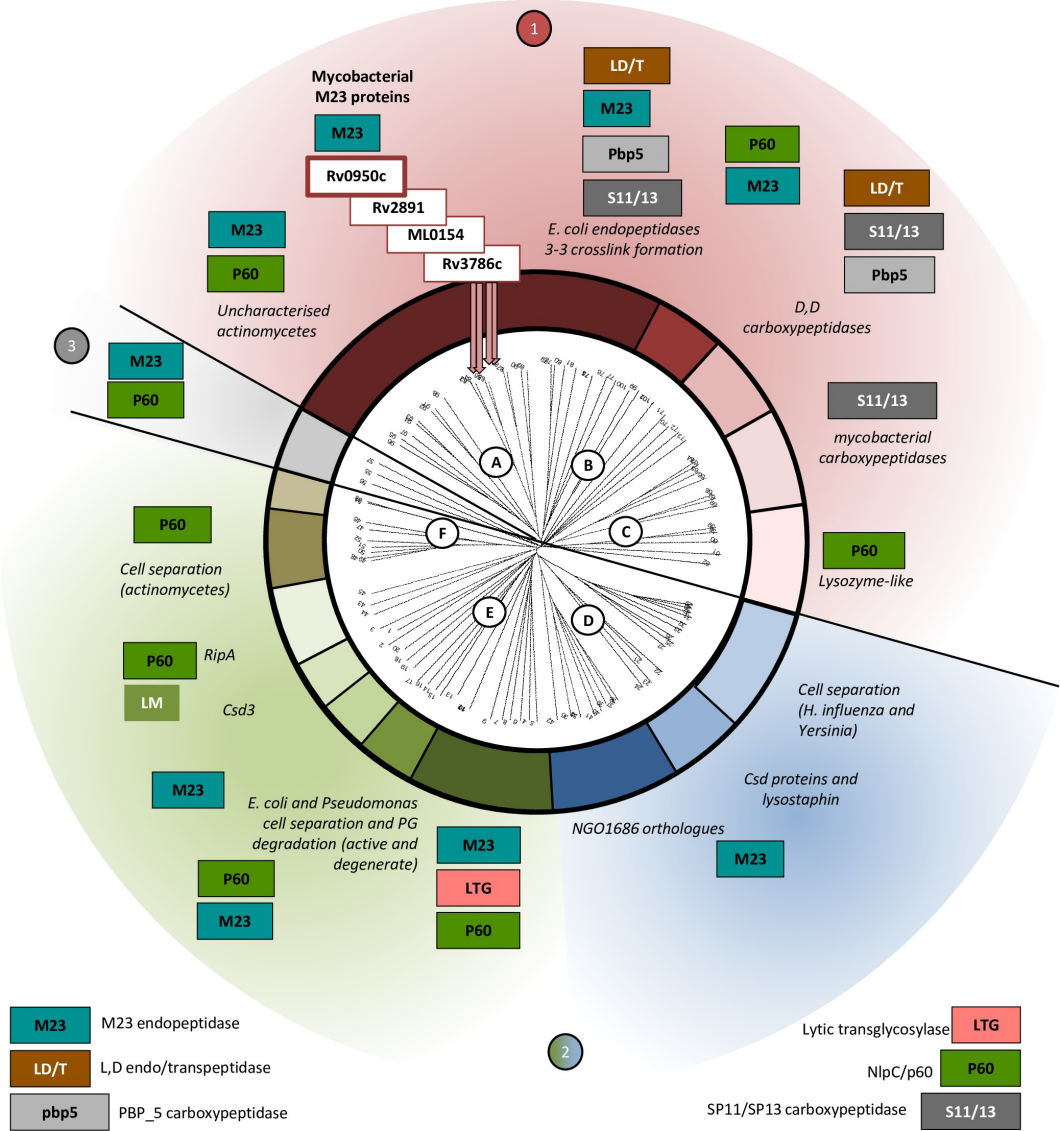
*M*. *tuberculosis* Rv0950c clusters with enzymes required for stem-peptide remodelling. Bacterial peptidoglycan hydrolases separate into three major clusters. The first, labelled 1—brown to light brown comprising sub-cluster A (uncharacterised homologues in actinomycetes, mycobacterial M23 and *E*. *coli* L,D-transpeptidases), B (*E*. *coli* carboxypeptidases) and C (mycobacterial carboxypeptidases and *B*. *subtilis* lysozyme-like NlpC/p60 enzymes). The second major cluster, labelled cluster 2—blue to green comprising sub-cluster D (*Helicobacter* and *Neisseria* cell-separation M23s and *S*. *simulans* lysostaphin), E (*E*. *coli* EnvC and NlpD orthologues, *M*. *tuberculosis* RipA and other cell separation enzymes) and F (cell separation NlpC/p60 enzymes of actinomycetes). An outlying cluster, labelled 3 –grey, comprises three proteins: *Neisseria meningitidis* M23 proteins of unknown function, and *M*. *avium paratuberculosis* MAP1272c, a PG-binding, degenerate NlpC/p60 protein.

Our bioinformatics analysis identified three M23-domain containing endopeptidase genes in the genome of *M*. *tuberculosis*, at loci Rv0950c, Rv2891 and Rv3786c, which are annotated as genes of unknown function. Identifying orthologues in the PHMMER databases and a survey of the literature allowed for construction of a phylogenetic tree, with some functional classifications (Fig 1). *M*. *tuberculosis* Rv0950c, Rv2891 and Rv3786c cluster alongside other mycobacterial orthologues, which form a greater sub-cluster with *E*. *coli* M23-domain YgeR and non-M23 endopeptidases, together with *E*. *coli* YcbB, AmpH, LdtD and PBP1b, some of which catalyse 3–3 cross-link formation in PG (Fig 1) [35–37]. This suggests that they may represent active peptidases. Neighbouring the *M*. *tuberculosis* M23 domain homologues are PG remodelling enzymes including *Corynebacterium jeikeium* PBP4, *E*. *coli* DacA (Fig 1 –sub-cluster A) and SP11 or SP13-domain mycobacterial carboxypeptidases [36,38–44] (Fig 1 –sub-cluster B). Other bacterial M23s formed an alternative, major cluster (Fig 1, labelled 2), including various cell division M23-endopeptidases and NlpC/p60 enzymes, such as the cell division factors, *E*. *coli* and *Haemophilus influenzae* EnvC and NlpD, the *Helicobacter pylori* cell shape determinant (Csd) homologues [45,46] and Staphylococcal lysins, lysostaphin and LytM [21,23,47]. Rv0950c is the only homologue of the three *M*. *tuberculosis* homologues that is conserved in *M*. *leprae* (S6 Table, S3 Fig), which retains the minimal genome required for virulence [48]. The synteny between Rv0950c and its counterpart in *M*. *leprae*, ML_0154c, is also conserved with proximal genes functioning in intermediary metabolism and cell wall processes (S3 Fig). In addition, Rv0950c is highly conserved in various mycobacterial species (S6 and S7 Tables) and collective consideration of all these features led us to focus on this gene for further characterisation.

### Rv0950c is predicted to be an active endopeptidase, expressed during growth in broth culture

**Fig 2:**
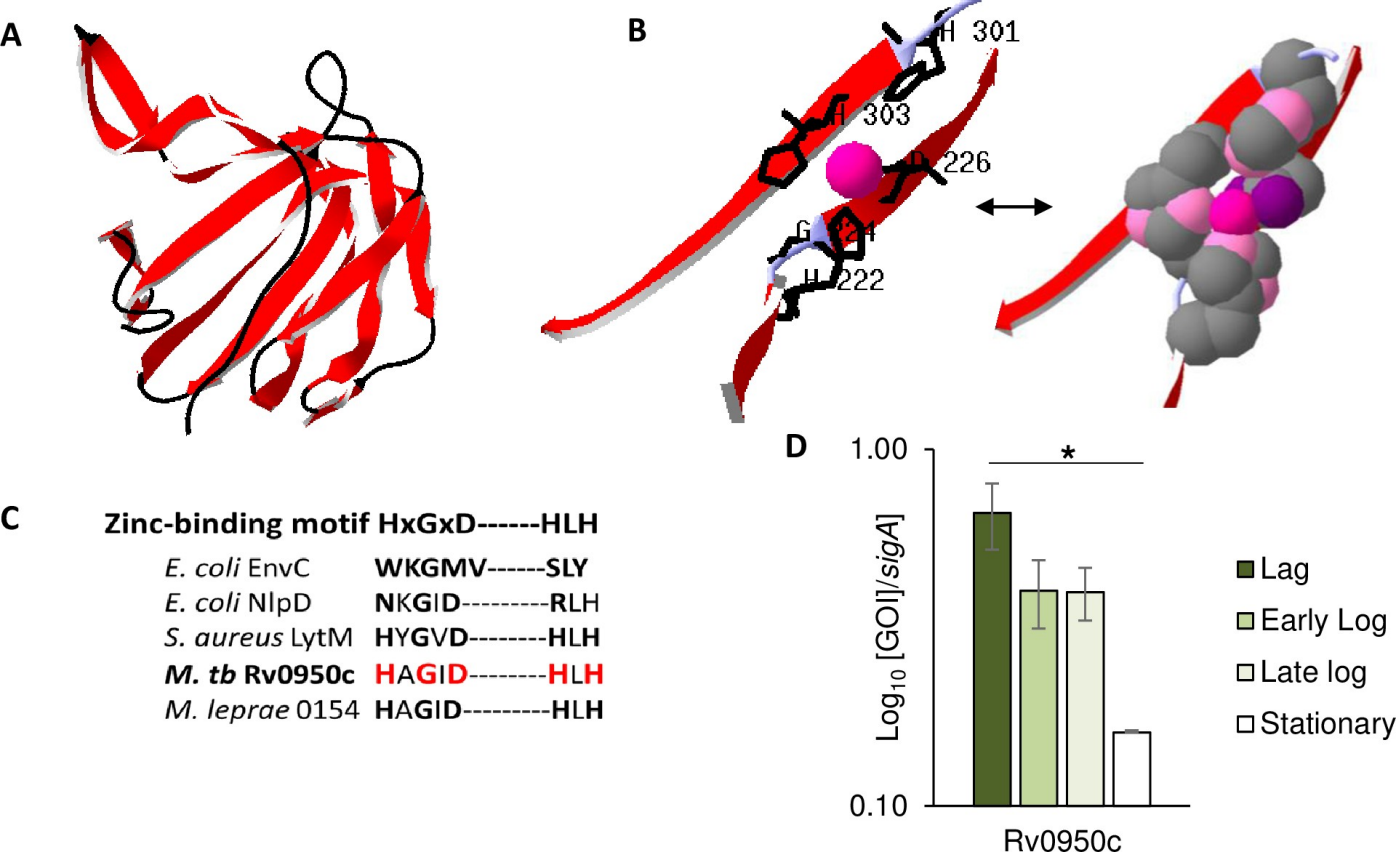
Homology modelling and transcriptomics suggest that *M*. *tuberculosis* Rv0950c is an active M23 endopeptidase expressed during growth. A) Rv0950c modelled with homology to *N*. *gonorrhoeae* Mpg (6muk); Red: β-sheets. B) The putative zinc-binding site of Rv0950c modelled to *V*. *cholerae* ShyA (6u2a.1). Zinc indicated in Magenta; Zinc-binding residues indicated in black. Space fill model predicts co-ordination of the Zn by nitrogen atoms (light pink) of H222, H303 and Oxygens (purple) of D226. C) Amino-acid sequence alignment of the M23 endopeptidase showing the conserved zinc-binding motif in the M23 endopeptidase domain of Rv0950c and the degenerate nature of this motif in *E*. *coli* homologues. D) Growth-phase dependent transcription of Rv0950c. Expression levels were measured by qPCR in three independent cultures and normalised to sigA. *p<0.02 by single-factor for Rv0950c ANOVA in Excel.

To assess the structure of Rv0950c, homology modelling was used with the Swiss Protein Database (http://swissmodel.expasy.org/repository/) [49–53]. For Rv0950c, the nearest structural orthologue was *Neisseria gonorrhoeae* Mpg, encoded by NG1686 (pdb id 6muk.1) [54] to which Rv0950c was modelled at a Global Model Quality Estimate (GMQE) of 0.27 and sequence identity of 40.16% (Fig 2A). *N*. *gonorrhoeae* Mpg catalyses degradation of 4–3 and 3–3 PG crosslinks and hydrolytic activity is dependent on an intact zinc-binding motif [55]. Zinc-binding capacity is an important predictor of PG hydrolytic activity as bacterial M23 endopeptidases appear to cluster either into PG degrading, active endopeptidases or degenerate sub-groups. Degenerate variants lack PG hydrolytic activity presumably due to the inability to bind zinc. The model generated against Mpg did not include Zinc as a ligand (Fig 2A). *V*. *cholerae* ShyA (pdb id 6u2a.1) [56] however had a similar homology score (GMQE = 0.26) to Mpg but with slightly less sequence homology, permitted a model for zinc co-ordination within the Rv0950c catalytic site (Fig 2B). Similar to Mpg, ShyA cleaves crosslinks in *V*. *cholerae* PG via an active M23 endopeptidase site [57]. Only the M23 domain of ShyA was included and not the Opacity-associated (OapA) domain. The modelling suggests that Rv0950c is similar to active M23-endopeptidases as it consists of the characteristic M23 β-sheet catalytic domain and retains the complete zinc-binding motif occurring in active *S*. *aureus* LytM, as opposed to degenerate residues of *E*. *coli* amidase interaction partners (EnvC and NlpD, Fig 2C). Assessment of gene expression during growth in broth culture revealed that Rv0950c exhibited a growth phase dependent transcription profile, with a reduction in expression upon entry into stationary phase (Fig 2D).

### Deletion of Rv0950c does not affect growth in broth culture but does cause a reduction in cell length of *M*. *tuberculosis*

**Fig 3:**
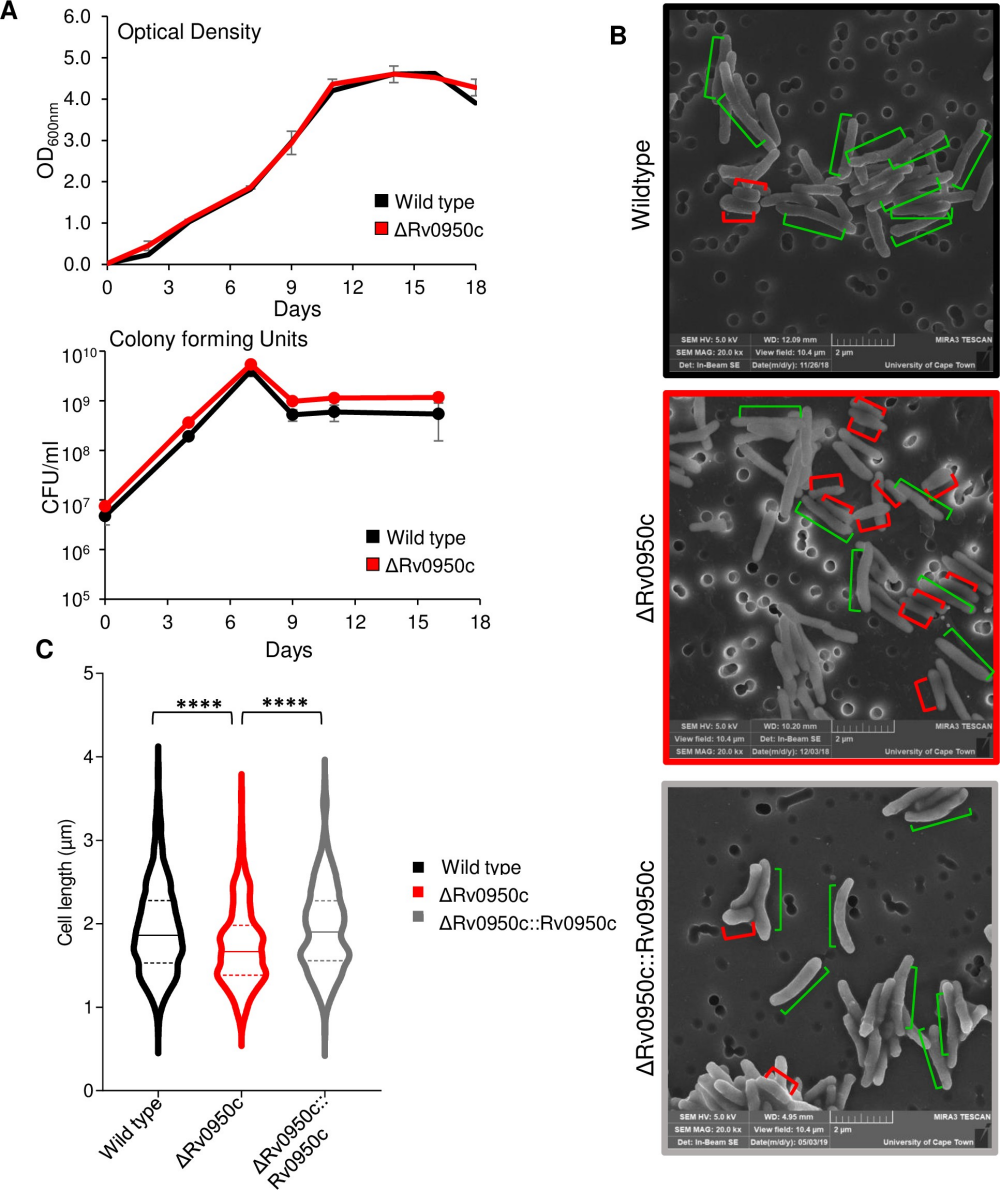
Deletion of Rv0950c does not affect growth but causes cell shortening in *M*. *tuberculosis*. A) Growth analysis of the ΔRv0950c mutant using optical density (upper panel) and colony forming unit (bottom panel). Deletion of Rv0950c resulted in no growth defects in broth culture B) Representative SEM micrographs of wild type, ΔRv0950c, and ΔRv0950c::Rv0950c genetically complemented derivative, emphasising normal cells of ~ 2 μm (green bars) and short cells of ~ 1 μm (red bars). Scale bar = 2 μm. C) Violin plot of cell lengths measured in ImageJ of 100 cells for each strain in triplicate. ****p<0.05E^-7^ for t-test calculated in Excel.

To further characterise the function of Rv0950c, a single, unmarked gene deletion strain was generated and the genotype confirmed by PCR and Southern blotting (S1 and S2 Figs). Genetic complementation was achieved by expressing Rv0950c from pTweety at the *lysU attB* site of the deletion mutant strain (S2 Fig). Deletion of Rv0950c did not cause any defects in growth (Fig 3A). To test the hypothesis that Rv0950c functions in cell wall metabolism, the effect of deletion thereof on cellular morphology was studied using SEM. We found that cells lacking Rv0950c formed rod-shaped bacilli, which were significantly shorter than wild type (*p* = 5.5E-08) and this phenotype could be reversed by complementation (*p* = 2.1E-10, comparing ΔRv0950c to complement; Fig 3B and 3C). These data suggest that Rv0950c is an M23-endopeptidase required for maintenance of cell length in *M*. *tuberculosis*.

### Differential FDAA incorporation in the Rv0950 deletion mutant

**Fig 4:**
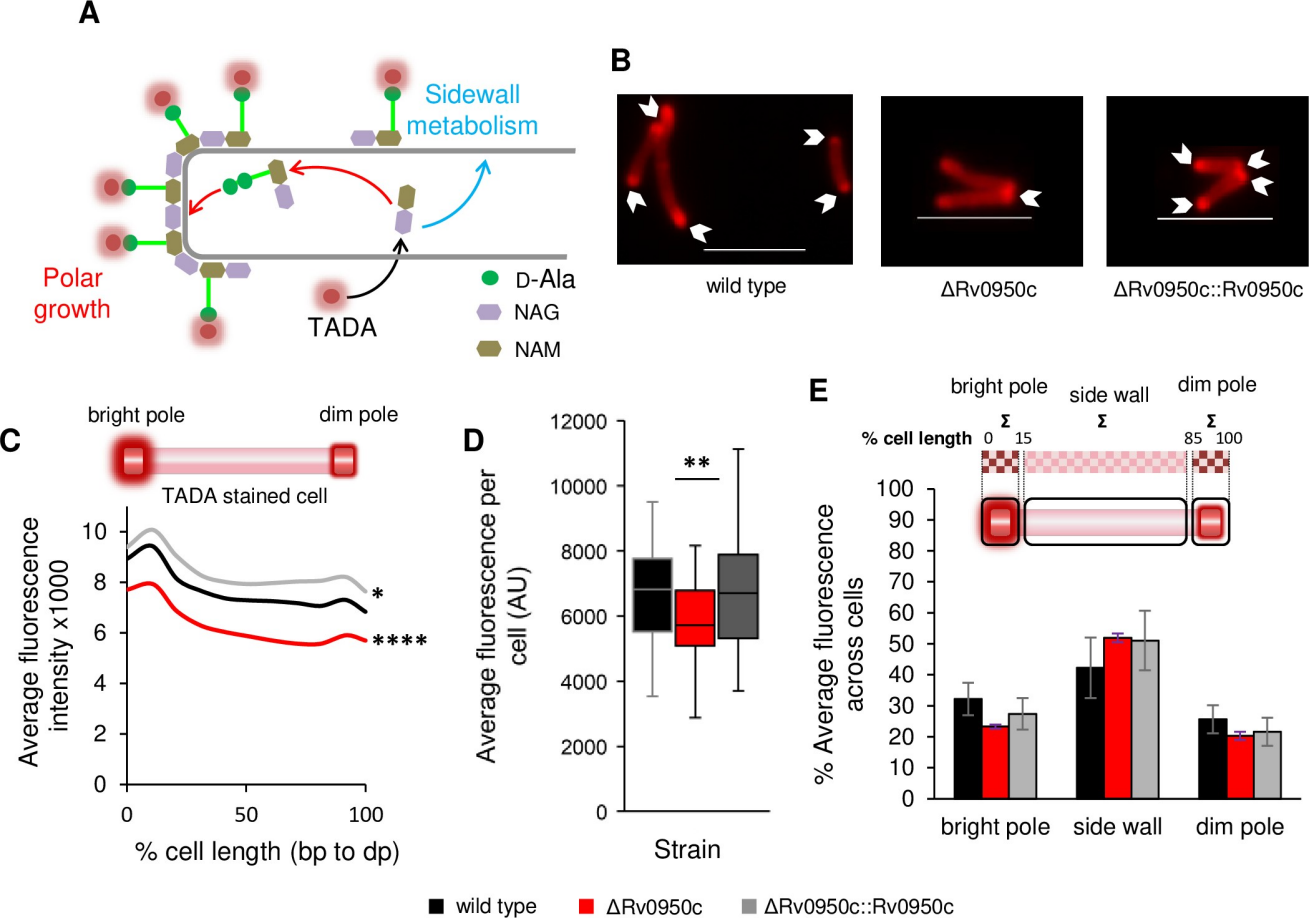
TADA incorporation in the Rv0950 deletion mutant. TADA incorporation was compared in *M*. *tuberculosis* wild type (WT, black), ΔRv0950c (red) and ΔRv0950c::Rv0950c (grey). A) Schematic diagram showing the mechanism of TADA incorporation reporting on polar growth (red) and side wall metabolism (turquoise), D-Ala: D-Alanine, NAG: N-acetylglucosamine, NAM: *N*-acetylmuramic acid. B) Representative fluorescence micrographs of stained cells. Scale bar = 5 μm. C) Fluorescence intensity profiles of *M*. *tuberculosis* labelled with TADA. 0% represents the brightest pole. Graph shows average of fluorescence intensity measured at 10% intervals of the cell length. ****p = 0.0008 and 4.09E^-6^ for mutant compared to wild type or complement respectively across the cell length. *p = 0.03 for wild type compared to complement. D) Average fluorescence intensity for each cell. **p = 0.0029 and 0.0049 comparing mutant to wild type and complement respectively. *p* values obtained by single factor ANOVA in Excel for three biological replicates. E) Average distribution of fluorescence between the bright pole (sum of fluorescence across the first 15% of the cell length), the side wall (sum of fluorescence between 16–84% of the cell length) and dim pole (sum of fluorescence from 85–100% of the cell length).

The development of FDAAs as fluorescent probes of PG building blocks has significantly advanced microscopic analysis of spatial PG synthesis and remodelling on the cell surface (Fig 4A) [5,6,58,59]. We sought to use these tools to determine spatial localization of PG biosynthesis and remodelling in the Rv0950 mutant strain. Uptake of TADA (a monopeptide FDAA) at the poles of *M*. *tuberculosis* cells, where nascent PG is localised, was observed after labelling for 30 minutes (Fig 4B). Cells lacking Rv0950c displayed a polar fluorescence distribution similar to that seen in the wild type, however there was a small but significant reduction in TADA fluorescence intensity across the cell when compared to the wild type and genetically complemented cells (*p* = 0.0008 and 4.09E-6 for mutant compared to wild type or complement respectively, Fig 4C; *p* = 0.002 and 0.004 comparing mutant to wild type and complement respectively, Fig 4D). Whilst this difference was statistically significant, the reduction in fluorescence intensity ranged between ca 10–15% across the lateral axis of the cell and could not be easily discerned from the fluorescence micrographs. As such, we consider this a marginal phenotypic defect, the physiological consequences of which are not yet known. We further compared spatial distribution of TADA incorporation among strains according to the method formulated by García-Heredia et al [6] and found no differences (Fig 4E).

**Fig 5:**
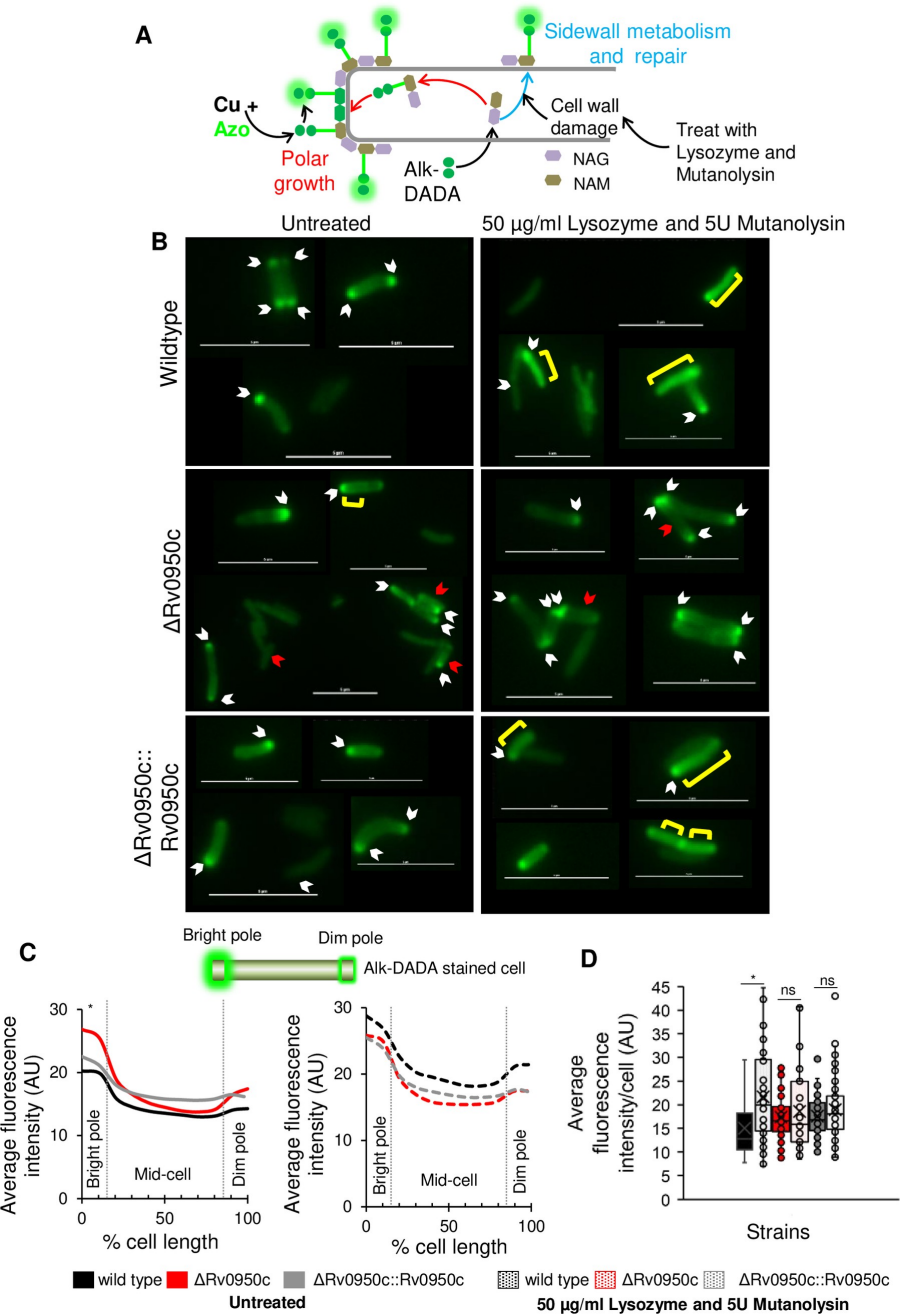
Incorporation alk-DADA upon the loss of Rv0950c. A) Schematic diagram showing alk-DADA incorporation during PG biosynthesis and detection via Cu-CAAC chemistry. When the PG backbone is damaged by lysozyme and mutanolysin treatment, alk-DADA is detected along the side wall therefore, this approach was used to assess changes in probe fluorescence in the ΔRv0950c deletion mutant. B) Representative fluorescence micrographs showing alk-DADA incorporation at the poles (white arrow) and the side wall (yellow bracket) of *M*. *tuberculosis* in untreated (left) and 50 μg/ml Lysozyme + 5 U Mutanolysin treated cells (right). Red arrows represent shorter cells observed for the mutant strain. Scale bar = 5 μm. C) Fluorescence intensity profiles of *M*. *tuberculosis* labelled with alk-DADA. Graph shows average fluorescence intensity measured at 10% intervals of the cell length. 0% represents the brightest pole. *p = 0.004 and 0.01 at 0% and 10% (bright pole) respectively comparing wild type to ΔRv0950c; *p = 0.01 and 0.02 at 0% and 10% (bright pole) respectively comparing complement to ΔRv0950c for student’s *t*-test. D) Average fluorescence intensity of *M*. *tuberculosis* labelled cells. *p = 0.0002 comparing wild type untreated to treated for students *t*-test; ns: Not significant.

We next assessed if uptake of a dipeptide probe was affected by loss of Rv0950c. We stained cells with alk-DADA and noted polar staining in both the wild type and ΔRv0950c mutant strains however, the mutant strain incorporated significantly more alk-DADA at the nascent pole (Fig 5B and 5C), with no change in average fluorescence across the remainder of the cell. In prior work, lysozyme and mutanolysin-induced damage of the PG backbone in *M*. *smegmatis* resulted in an increased alk-DADA signal at the side-wall, presumably as a result of nascent PG insertion at sites of PG damage [6]. We tested this in *M*. *tuberculosis* and similarly found that cell wall damage resulted in increased sidewall labelling with alk-DADA in the wild type, which also manifested as an increase in average fluorescence intensity per cell (Fig 5B and 5D, there was an increase in labelling from 14,6 ± 2,64 AU [untreated] to 22,3 ± 4,10 AU [treated], p = 0.0002). In contrast, this effect was abrogated in the ΔRv0950c where there was no significant difference in sidewall labelling or average fluorescence intensity upon cell wall damage (Fig 5B and 5D, 17,4 ± 0,53 AU [untreated] to 16,4 ± 3,48 AU [treated]). Genetic complementation partially resolved this defect (Fig 5D) as fluorescence intensity did increase upon treatment (17,1 ± 1,59 AU [untreated] to 18,2 ± 0.48 AU [treated]) but this difference was not statistically significant.

### Loss of Rv0950c does not affect cell wall permeability, susceptibility to antibiotics/oxidative stress, alter colony morphology, nor does it affect intracellular survival

**Fig 6:**
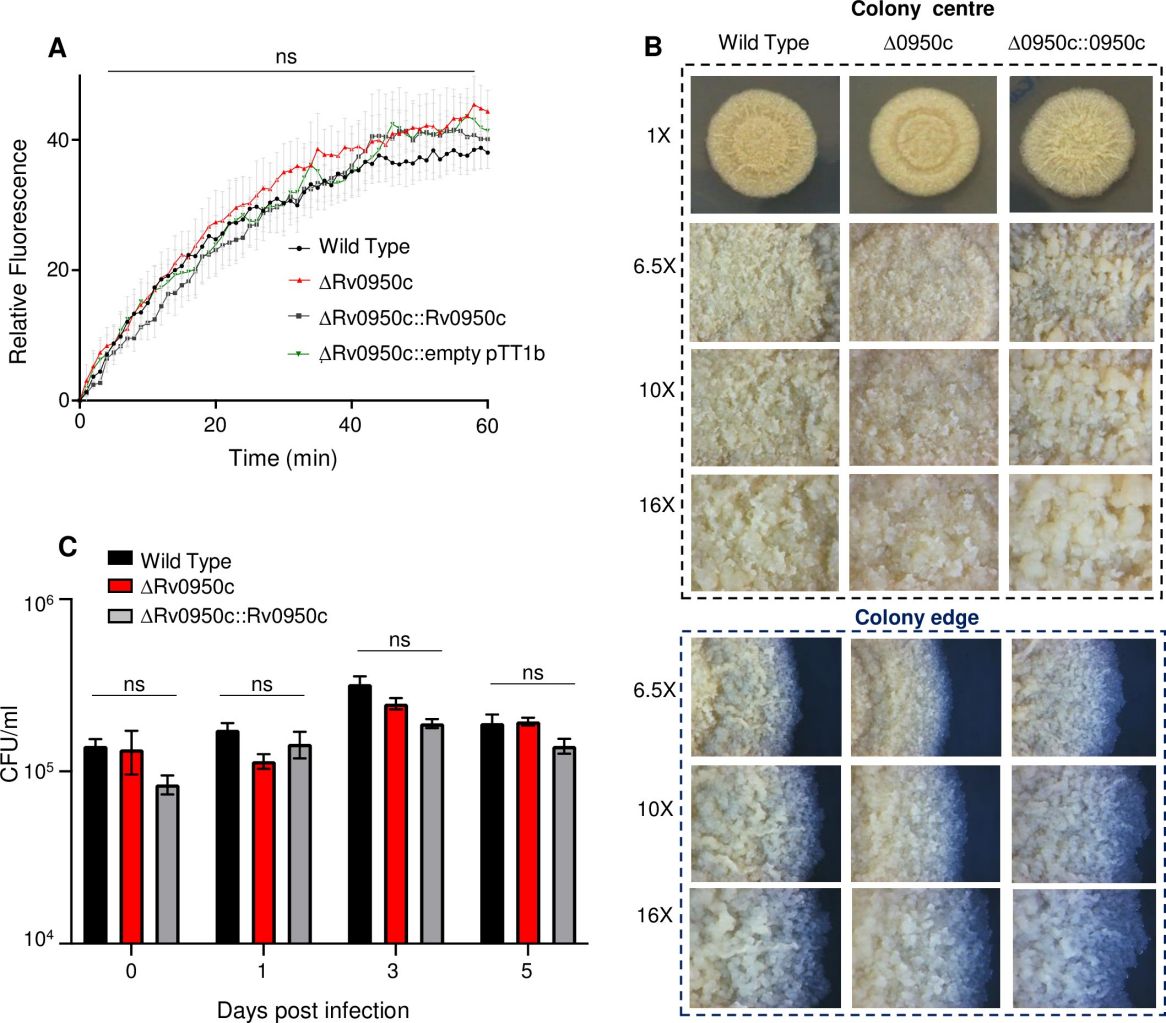
Assessment of cell wall permeability, colony morphology and intracellular survival of the Δ0950c mutant. (A) Ethidium bromide uptake as a measure of cell wall permeability. A plate-based fluorescence readout over 60 min incubation with EtBr (0.25 μg/ml) was used. Data was averaged from duplicate readings of three biological replicates for each strain. As ethidium bromide enters the cell, fluorescence is increased (B) Morphology of colonies from the Δ0950c mutant. Cultures were grown to mid-log phase (OD600_nm_ ~ 0.5) in 7H9 media supplemented with OADC and Tween. Ten microliters of each were spotted onto 7H11 media and incubated at 37°C for 21 days. Images were captured at magnifications shown to compare shape and structures on colonies (centre and edge). (C) Intracellular survival of the wild type, Δ0950c mutant and genetically complemented strains. The mutant, wild type and genetically complemented derivative were infected in activated THP1 cells and survival monitored by enumerating colony forming units (CFU/ml) over a period of 5 days. ns: Not significant.

As Rv0950c appears to be phylogenetically related to cell wall remodelling enzymes, we hypothesized that deletion thereof would affect cell wall permeability. Previously, we assessed cell wall permeability in a PG-amidase defective mutant by monitoring diffusion of ethidium bromide into the cell, using fluorescence to monitor accumulation [9]. Using a similar approach, we noted no difference in permeability of the ΔRv0950c mutant when compared to the wild type (Fig 6A). In our prior work with cell wall remodelling enzymes such as lytic transglycosylases, we noted marked differences in the appearance of colonies on agar plates and susceptibility to antibiotics, presumably due to changes in cell wall structure/function [60–62]. To determine if similar effects prevailed in the ΔRv0950c mutant, we assessed colony morphology on Middlebrook agar and noted no differences when compared to the wild type strain (Fig 6B). We also tested susceptibility to cell wall targeting antibiotics/agents and did not find any increased susceptibility with the ΔRv0950c mutant (S8 Table). Next, given that Mpg in *N*. *gonorrhoeae* is required for resistance to oxidative stress, we tested the susceptibility of the ΔRv0950c mutant to menadione, a potent generator of oxidative stress in mycobacteria, but found no differential susceptibility when compared to the wild type (S8 Table). Finally, we assessed intracellular survival of the ΔRv0950c mutant in activated THP1 macrophages and found no differences when compared to the wild type and genetically complemented derivative (Fig 6C).

## Discussion

M23 endopeptidases represent a class of highly versatile enzymes required for growth and/or virulence of pathogenic bacteria [21,45,47,63] however, these proteins remain unexplored in *M*. *tuberculosis*. Herein, we studied the function of Rv0950c, a previously uncharacterised gene and demonstrated that it is dispensable for growth in axenic culture but plays a role in regulating cell length and to a limited extent, PG remodelling in *M*. *tuberculosis*. Bioinformatics and phylogeny analyses suggests it is highly conserved in structure but functionally divergent from non-mycobacterial M23 endopeptidases. Rv0950c instead clusters with enzymes required for PG crosslink formation and transpeptidation and is structurally similar to catalytically active M23 endopeptidases in *N*. *gonorrhoeae* [55] and *V*. *cholerae* [57].

Loss of Rv0950 led to a reduction in cell length and changes in uptake of FDAAs in response to cell wall damage. Two main routes of FDAA incorporation have been reported in mycobacteria: intracellular PG biosynthesis and extracellular D,D and L,D transpeptidation [6]. The dipeptide, alk-DADA, is incorporated intracellularly during PG biosynthesis and flipped out of the cell incorporated in lipid II, representing uncrosslinked PG [6]. Therefore, the increased alk-DADA fluorescence at the nascent pole of ΔRv0950c cells could suggest a role for this enzyme in PG remodelling. In *E*. *coli* and *B*. *subtilis*, enhanced fluorescence of cell wall probes was observed when depletion of D,D-carboxypeptidases prevented crosslink degradation [64,65]. In our prior work, we observed that depletion of an essential D,D-carboxypeptidase in *M*. *smegmatis* resulted in the loss of bi-polar labelling and an increase in mono-polar labelling [40]. It is possible that an increased alk-DADA signal at the nascent pole reflects dysregulation of D,D-carboxypeptidase activity, resulting in reduced removal of the terminal D-ala residue however, this requires further investigation.

The reduced uptake of TADA in the ΔRv0950c mutant also suggests that PG biosynthesis and turnover appears to be perturbed however, this effect was marginal. In *E*. *coli* and *B*. *subtilis*, enhanced fluorescence of cell wall probes was observed in response to inhibition of cross-link remodelling [64,65]. Unlike *E*. *coli* and *B*. *subtilis*, actinomycetes have a characteristically high abundance of 3–3 cross linked PG [66]. Ldts catalyse 3–3 crosslink formation in mycobacteria and deletion of all six homologues in *M*. *smegmatis* resulted in reduced FDAA incorporation [5]. Herein, the overall reduction in TADA incorporation in *M*. *tuberculosis* upon deletion of Rv0950c could suggest higher levels of un-crosslinked PG.

Cell shape maintenance is also an important component of the stress response in mycobacteria [67]. In a non-replicating model, *M*. *tuberculosis* exhibited cell shortening and even loss of the cell wall [68]. Similar phenotypes were also observed in other actinomycetes [69]. Under stress conditions, metabolism of cell signaling molecules regulated elongation and cell division, thus maintaining cell shape [70]. For example, host-derived reactive nitrogen species repressed Rv0950c expression [71]. This appears to contradict a study wherein Rv0950c expression was unchanged during active mouse infection, and downregulated during immune suppression-induced *M*. *tuberculosis* reactivation [72]. Notably, the *N*. *gonorrhoeae* Mpg protein, to which Rv0950c was modelled here, is required for virulence and resistance to hydrogen peroxide [55,73], however, in this study we noted no increased susceptibility with the ΔRv0950c mutant in response to oxidative stress. This suggests the possibility that the mycobacterial M23-domain containing peptidases have diverged in function from other previously described counterparts. As cell length maintenance is required for adaptation to stress, we explored the role of Rv0950c as a contributor to intracellular survival of *M*. *tuberculosis* but found no difference in survival of the mutant in activated THP1 macrophages. Collectively, these data suggest genetic and functional redundancy in M23-domain-containing proteins, given the presence of two more putative M23 endopeptidases in the *M*. *tuberculosis* genome. Assessment of mutants with deletions in multiple M23-domain proteins may yield more pronounced phenotypic defects.

Herein, we describe the characterisation of an M23 domain encoding gene in *M*. *tuberculosis*. The high conservation of these proteins in mycobacteria, together with a potential role in morphological and cell wall homeostasis, suggest that they merit further study as potential regulators of cell growth and division in actinobacteria.

## Supporting information

S1 Fig Confirmation of Rv0950c deletion in the *M*. *tuberculosis* genome by PCR and phenotypic screening for suicide vector loss. A) Phenotypic screening for suicide vector loss in the Rv0950 deletion mutant. Wild type, SCO and the mutant strains were spotted on Middlebrook 7H11 with and without kanamycin. The suicide vector confers kanamycin resistance, which should be lost during the second crossover event to generate the mutant. As expected the mutant and wild type are not resistant to kanamycin. Two independent replicates are shown. B) Schematic diagram of genomic regions of the wild-type and mutant strains showing primer binding sites. Also shown is an agarose gel of the PCR fragments that result from using these primers. SCO–single crossover strain.
(PDF)

S2 Fig Confirmation of Rv0950c deletion in the *M*. *tuberculosis* genome by Southern blotting and gene expression analysis. A) Schematic diagram of genomic regions of the wild-type (WT), mutant and complement strains showing Rv0950c probe binding (green) to respective *Bgl*I fragments. *M*. *tuberculosis* genes indicated in black, Rv0950c indicated in red, pTTP1b phage vector genes indicated in grey. Deletion of Rv0950c has been genetically complemented at the *lysU-attB* integration site. Boxes show *attB* and *attP* regions of homology for pTTP1b integration. B) Expected *Bgl*I fragments complementary to the Rv0950c probe for each strain. C) Southern blot 1) MWM IV, 2) WT, 3) ΔRv0950c, 4) ΔRv0950c::Rv0950C. MWM: Digested Lambda phage DNA marker. D) RT-qPCR to confirm loss of Rv0950c transcription in the ΔRv0950c strain and restoration of transcription in the ΔRv0950c::Rv0950C (lysU::pTTP1bRv0950c) complemented strain.
(PDF)

S3 Fig Conserved genetic synteny between Rv0950c and the only orthologue in *M*. *leprae*. *M*. *leprae* represents the most reduced genome among the mycobacteria. Representation of genetic neighbourhoods displayed by Mycobrowser (https://mycobrowser.epfl.ch) illustrating high degree of conservation in the arrangement of upstream and downstream genes.
(PDF)

S1 Table Strains of *M*. *tuberculosis* used and generated in this study. (PDF)

S2 Table Primer sequences for qPCR. (PDF)

S3 Table List of bacterial plasmid vectors used in this study. (PDF)

S4 Table Primers for amplification of cloning inserts for knockout of generation of a genetically complemented strain. (PDF)

S5 Table Primer sequences for genotyping gene deletion and complementation. (PDF)

S6 Table Mycobacterial expansion of M23 endopeptidases highlights Rv0950c as the most conserved orthologue in *M*. *tuberculosis*. Direct orthologues occur in the same row. Orthologues were identified in the PHMMER database [31].
(PDF)

S7 Table Distribution of Rv0950c orthologues in mycobacterial species. Data collected from PHMMER, pFAM and Mycobrowser [28,30,31].
(PDF)

S8 Table Broth microdilution determination of MIC (μg/ml) of cell-wall targeting antibiotics, lysozyme and ROS-generating compounds*. (PDF)

S1 Raw images (PDF)
