# Supplementary material for: Characterisation of a putative M23-domain containing protein in Mycobacterium tuberculosis
Source: PLoS One. 2021 Nov 16;16(11):e0259181. doi: 10.1371/journal.pone.0259181 (PMC8594824; doi:10.1371/journal.pone.0259181)
Supplement: S1 Fig — A) Phenotypic screening for suicide vector loss in the Rv0950 deletion mutant. Wild type, SCO and the mutant strains were spotted on Middlebrook 7H11 with and without kanamycin. The suicide vector confers kanamycin resistance, which should be lost during the second crossover event to generate the mutant. As expected the mutant and wild type are not resistant to kanamycin. Two independent replicates are shown. B) Schematic diagram of genomic regions of the wild-type and mutant strains showing primer binding sites. Also shown is an agarose gel of the PCR fragments that result from using these primers. SCO–single crossover strain. (PDF) [file pone.0259181.s001.pdf]

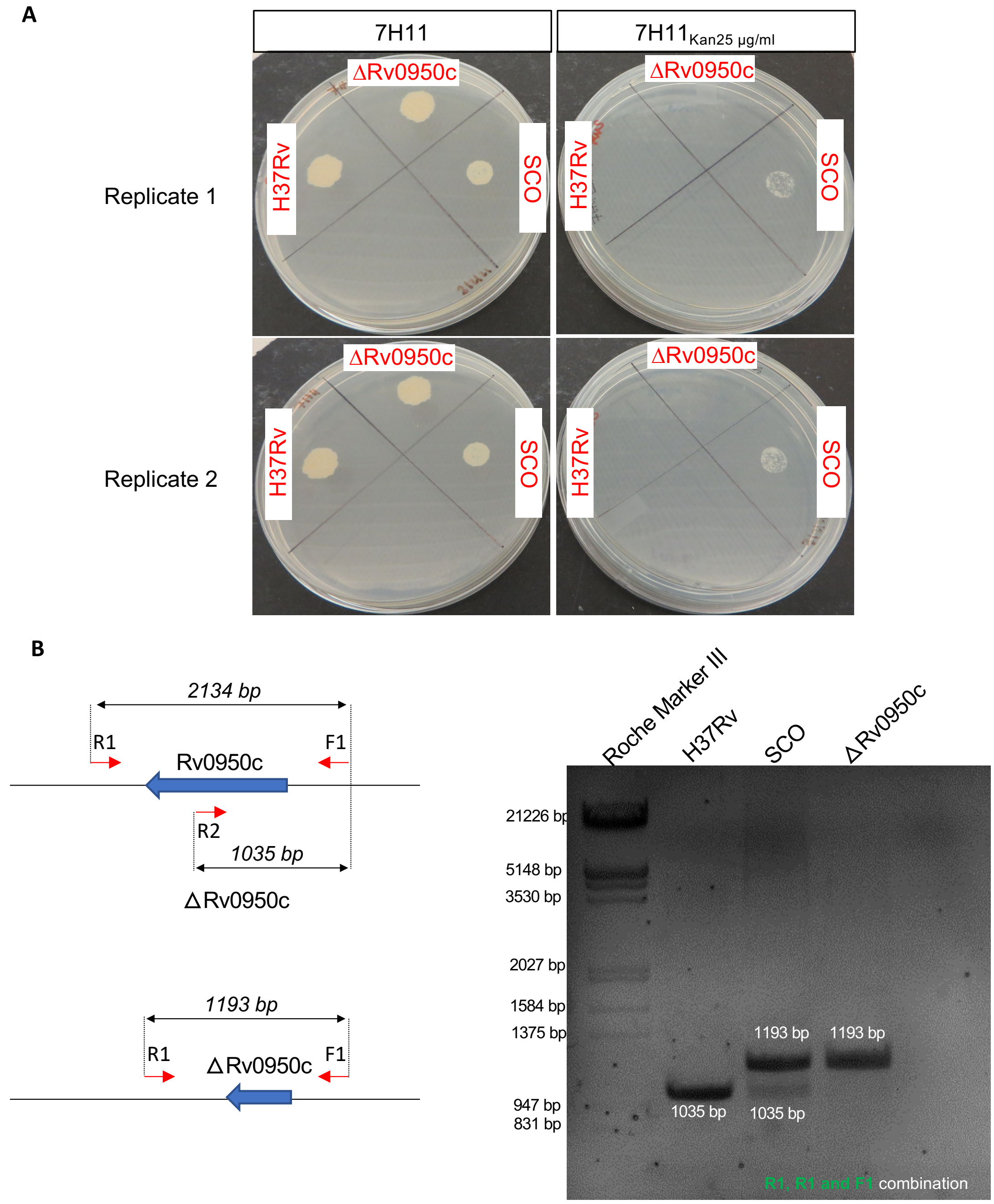

Expected sizes: All three primers R1, R2 and F1 are used in one reaction.

- In the Wild type, two products are possible, the result of PCR between R1 and F1 (2134 bp) and R2 and F1 (1035 bp). Generally, in PCR, the smaller product is favoured and in this case, we observed the 1035 bp fragment
- In the mutant, the priming site for R2 has been deleted and the expected product is 1193 bp
- In the SCO, fragments diagnostic for both the wild type (1035 bp) and the mutant (1193 bp) are expected.

Figure S1. Confirmation of Rv0950c deletion in the *M. tuberculosis* genome by PCR and phenotypic screening for suicide vector loss. A) Phenotypic screening for suicide vector loss in the Rv0950 deletion mutant. Wild type, SCO and the mutant strains were spotted on Middlebrook 7H11 with and without kanamycin. The suicide vector confers kanamycin resistance, which should be lost during the second crossover event to generate the mutant. As expected the mutant and wild type are not resistant to kanamycin. Two independent replicates are shown. B) Schematic diagram of genomic regions of the wild-type and mutant strains showing primer binding sites. Also shown is an agarose gel of the PCR fragments that result from using these primers. SCO – single crossover strain.
