# Supplementary material for: Characterisation of a putative M23-domain containing protein in Mycobacterium tuberculosis
Source: PLoS One. 2021 Nov 16;16(11):e0259181. doi: 10.1371/journal.pone.0259181 (PMC8594824; doi:10.1371/journal.pone.0259181)
Supplement: S2 Fig — A) Schematic diagram of genomic regions of the wild-type (WT), mutant and complement strains showing Rv0950c probe binding (green) to respective BglI fragments. M. tuberculosis genes indicated in black, Rv0950c indicated in red, pTTP1b phage vector genes indicated in grey. Deletion of Rv0950c has been genetically complemented at the lysU-attB integration site. Boxes show attB and attP regions of homology for pTTP1b integration. B) Expected BglI fragments complementary to the Rv0950c probe for each strain. C) Southern blot 1) MWM IV, 2) WT, 3) ΔRv0950c, 4) ΔRv0950c::Rv0950C. MWM: Digested Lambda phage DNA marker. D) RT-qPCR to confirm loss of Rv0950c transcription in the ΔRv0950c strain and restoration of transcription in the ΔRv0950c::Rv0950C (lysU::pTTP1bRv0950c) complemented strain. (PDF) [file pone.0259181.s002.pdf]

A

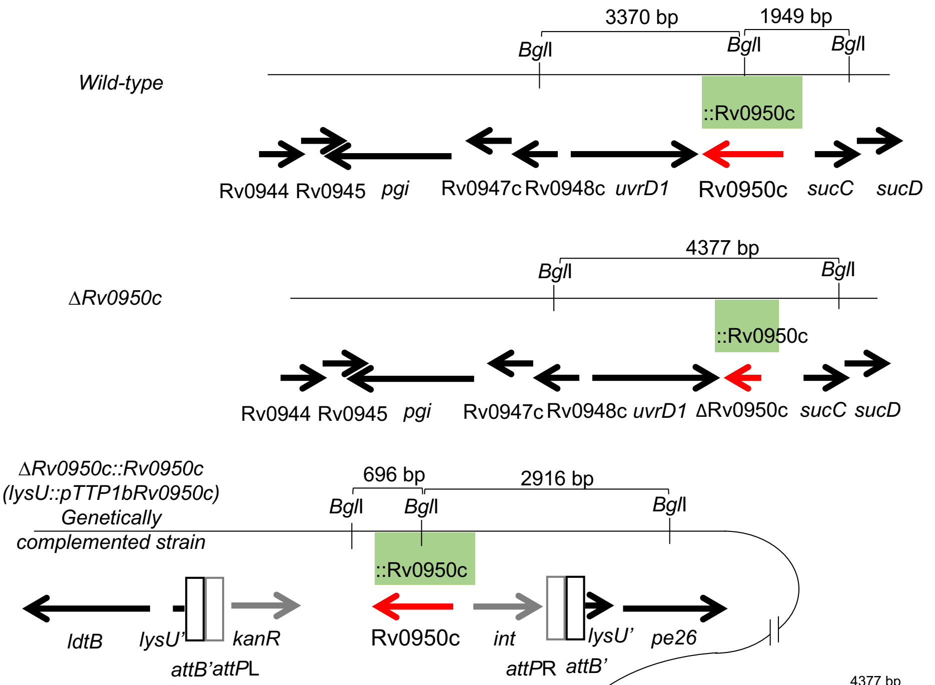

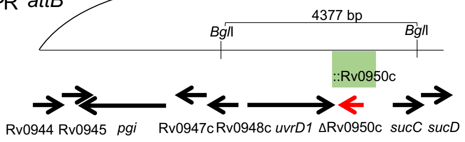

B

Strain	***BglI* fragments (bp)**
Wild-type (H37RvS)	1949, 3370
ΔRv0950c	4377
ΔRv0950c::Rv0950c (lysU::pTTP1bRv0950c)	696, 2916, 4377

C

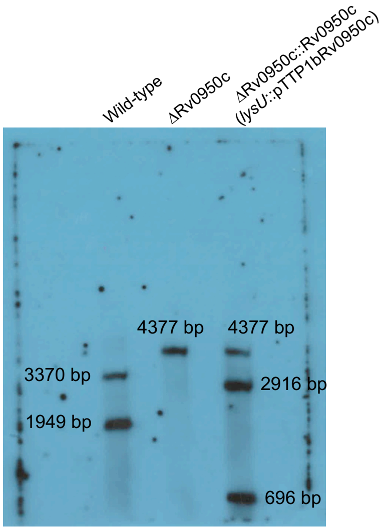

D

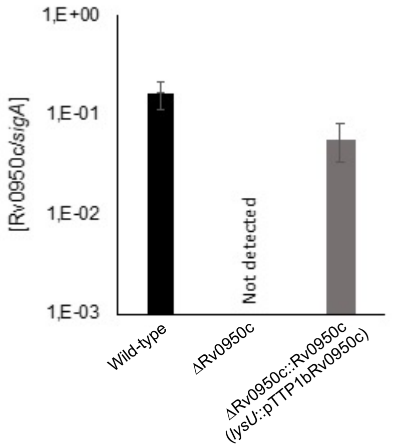

Fig. S2. Confirmation of Rv0950c deletion in the *M. tuberculosis* genome by Southern Blotting and gene expression analysis. A) Schematic diagram of genomic regions of the wild-type (WT), mutant and complement strains showing Rv0950c probe binding (green) to respective *Bgl*I fragments. *M. tuberculosis* genes indicated in black, Rv0950c indicated in red, pTTP1b phage vector genes indicated in grey. Deletion of Rv0950c has been genetically complemented at the *lysU-attB* integration site. Boxes show *attB* and *attP* regions of homology for pTTP1b integration. B) Expected *Bgl*I fragments complementary to the Rv0950c probe for each strain. C) Southern blot 1) MWM IV, 2) WT, 3) ΔRv0950c, 4) ΔRv0950c::Rv0950C. MWM: digested Lambda phage DNA marker. D) RT-qPCR to confirm loss of Rv0950c transcription in the ΔRv0950c strain and restoration of transcription in the ΔRv0950c::Rv0950C (lysU::pTTP1bRv0950c) complemented strain.
