# Supplementary material for: Characterisation of a putative M23-domain containing protein in Mycobacterium tuberculosis
Source: PLoS One. 2021 Nov 16;16(11):e0259181. doi: 10.1371/journal.pone.0259181 (PMC8594824; doi:10.1371/journal.pone.0259181)
Supplement: S3 Fig — M. leprae represents the most reduced genome among the mycobacteria. Representation of genetic neighbourhoods displayed by Mycobrowser (https://mycobrowser.epfl.ch) illustrating high degree of conservation in the arrangement of upstream and downstream genes. (PDF) [file pone.0259181.s003.pdf]

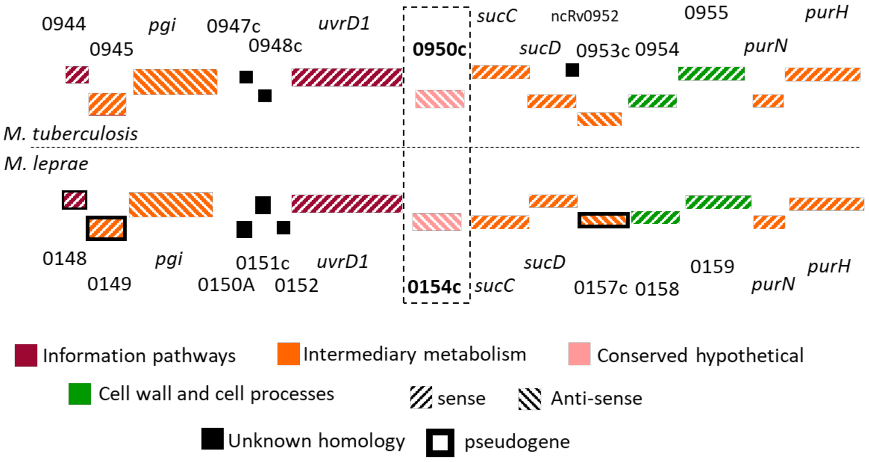

Figure S3. Conserved genetic synteny between Rv0950c and the only orthologue in *M. leprae*. *M. leprae* represents the most reduced genome among the mycobacteria. Representation of genetic neighbourhoods displayed by Mycobrowser [1] (https://mycobrowser.epfl.ch/) illustrating high degree of conservation in the arrangement of upstream and downstream genes.

References

1. Kapopoulou A, Lew JM, Cole ST. The MycoBrowser portal: A comprehensive and manually annotated resource for mycobacterial genomes. Tuberculosis. 2011;91(1):8-13. doi: 10.1016/j.tube.2010.09.006.
