# Supplementary material for: Characterisation of a putative M23-domain containing protein in Mycobacterium tuberculosis
Source: PLoS One. 2021 Nov 16;16(11):e0259181. doi: 10.1371/journal.pone.0259181 (PMC8594824; doi:10.1371/journal.pone.0259181)
Supplement: S1 Table — (PDF) [file pone.0259181.s004.pdf]

Table S1. Strains of *M. tuberculosis* used and generated in this study.

Strain	Description	Reference
**H37Rv(s)**	*M. tuberculosis* wild-type parent strain	Lab stock
**H37Rv(s)ΔRv0950c**	Derivative of H37Rv(s) carrying an unmarked, in-frame deletion of region 1060692-1061627 of Rv0950c	This study
**H37Rv(s)ΔRv0950c *lysU*::pTTp1b-^{NP}Rv0950c**	Derivative of the Rv0950c deletion mutant strain complemented with Rv0950c and the putative native promotor from the pTTp1b backbone at the *attB* region of *M. tuberculosis lysU*, Km^R	This study
