# Supplementary material for: Characterisation of a putative M23-domain containing protein in Mycobacterium tuberculosis
Source: PLoS One. 2021 Nov 16;16(11):e0259181. doi: 10.1371/journal.pone.0259181 (PMC8594824; doi:10.1371/journal.pone.0259181)
Supplement: S2 Table — (PDF) [file pone.0259181.s005.pdf]

Table S2. Primer sequences for qPCR.

Target	Sequence	Co-ordinates	Product size (bp)
**Rv0950c**	F: TTATCGATGGGGTGTACTGCA	1060990-1061010	133 (F + R1); 129 (F + R2)
	R1: CAGCAGCTTGACCCACATTC	1060878-1060897	
	R2: AGCTTGACCCACATTCCGTA	1060882-1060901	
sigA	F: TGCAGTCGGTGCTGGACAC	3019213-3019231	195
	R: CGCGCAGGACCTGTGAGCGG	3019388- 3019407	
