# Supplementary material for: Characterisation of a putative M23-domain containing protein in Mycobacterium tuberculosis
Source: PLoS One. 2021 Nov 16;16(11):e0259181. doi: 10.1371/journal.pone.0259181 (PMC8594824; doi:10.1371/journal.pone.0259181)
Supplement: S3 Table — (PDF) [file pone.0259181.s006.pdf]

Table S3. List of bacterial plasmid vectors used in this study.

Vector	Description	Selection	Reference
pBlueScript	Cloning vector with multiple cloning site inside *lacZ* for blue-white selection	Amp^R, *lacZ*	[1]
pBlue09US	Rv0950c Upstream region blunt cloned into pBlueScript at the *Sma*I site	Amp^R, *lacZ*	This study
p2Nil	Suicide vector lacking a mycobacterial *ori*	Km^R	[2]
p2NilΔRv0950c	p2Nil with fusion of Rv0950c upstream and downstream regions for suicide vector-mediated Rv0950c deletion	Km^R	This study
pGOAL17	Vector carrying *Pac*I-flanked *lac_sac* selectable markers to be sub-cloned into p2Nil vectors	Amp^R, *lacZ, sacB*	[2]
p2NilΔ09SV	p2NilΔRv0950c suicide vector with pGOAL-17 derived *lac_sac* genes	Km^R, *lacZ*, *sacB*	This study
pTweety (pTTP1b)	Mycobacterial, phage derived, integration vector for genetic complementation at the *attB* region of *M. tuberculosis lysU*	Km^R	[3]
pT09	pTweety with Rv0950c and putative native promotor for genetic complementation	Km^R	This study

1. Alting-Mees MA, Short JM. pBluescript II: gene mapping vectors. Nucleic Acids Res. 1989;17(22):9494. Epub 1989/11/25. doi: 10.1093/nar/17.22.9494. PubMed PMID: 2555794; PubMed Central PMCID: PMCPMC335171.
2. Parish T, Stoker NG. Use of a flexible cassette method to generate a double unmarked *Mycobacterium tuberculosis* tlyA plcABC mutant by gene replacement. Microbiology. 2000;146:1969-75. doi: 10.1099/00221287-146-8-1969.
3. Pham TT, Jacobs-Sera D, Pedulla ML, Hendrix RW, Hatfull GF. Comparative genomic analysis of mycobacteriophage Tweety: Evolutionary insights and construction of compatible site-specific integration vectors for mycobacteria. Microbiology. 2007;153(8):2711-23. doi: 10.1099/mic.0.2007/008904-0.
