# Supplementary material for: Characterisation of a putative M23-domain containing protein in Mycobacterium tuberculosis
Source: PLoS One. 2021 Nov 16;16(11):e0259181. doi: 10.1371/journal.pone.0259181 (PMC8594824; doi:10.1371/journal.pone.0259181)
Supplement: S4 Table — (PDF) [file pone.0259181.s007.pdf]

**Table S4.** Primers for amplification of cloning inserts for knockout of generation of a genetically complemented strain

| Target | Sequence | Restriction site | Genomic co-ordinates | $T_A$ | Product size |
|---|---|---|---|---|---|
| **Rv0950c upstream** | F: gccgcc*aagctt*TTGACGAACACGCTCTTGAC | *HinD*III | 1062885-1062904 | 64 | 1301 |
| | R: gcgcgc*agatct*ATCGCGAGGTGTGCGAAT | *Bgl*II | 1061628-1061645 | | |
| **Rv0950c downstream** | F: gcgcgcagatctAAGCGGGGACTTAGCGTCGGCAATTA | *Bgl*II | 1060666-1060691 | 76 | 1423 |
| | R: gccgcc*ctgcag*GCTATGTCGCCGACAACGAGCAC | *Pst*I | 1059293-1059315 | | |
| **Rv0950c complement** | F: gcgcgc*tctaga*GTCTTCGCTCGGCTTACT | *Xba*I | 1061937-1061954 | 65 | 1360 |
| | R: gccgccggtaccTTCTCGCCCTGAGAACAC | *Acc65*I | 1060619-1060636 | | |

Lower case text, no italics indicate the GC clamp. Lower case, underlined italics denote restriction sites. $T_A$=annealing temperature
