# Supplementary material for: Characterisation of a putative M23-domain containing protein in Mycobacterium tuberculosis
Source: PLoS One. 2021 Nov 16;16(11):e0259181. doi: 10.1371/journal.pone.0259181 (PMC8594824; doi:10.1371/journal.pone.0259181)
Supplement: S5 Table — (PDF) [file pone.0259181.s008.pdf]

Table S5. Primer sequences for genotyping mutants and genetic complementation.

Target	Sequence	T_A	co-ordinates
**ΔRv0950c (Genotyping)**	F1: CTTGGCCCAGACGACGAAGA	64	1059925-1059944
	F2: CAGGAGCTCATCGACTGGC		1060288-1060306
	R1: TTGGCACCCTCGGCTGTGTC		1062039-1062058
	R2: ACCCCGATTTATGCGGTGTCC		1060938-1060958
**pTweety Km^R**	F: ATTCAACGGGAAACGTCTTG	55	
	R: ATTCCGACTCGTCCAACATC		
**Rv0950+promoter (Genetic complementation)**	F1: gcgcgc*tctaga*GTCTTCGCTCGGCTTACT	60	1061937-1061954
	R1: gccgcc*actagt*ACCGGTGTAATTGCCGAC		1060659-1060676
	R2: gccgcc*ggtacc*TTCTCGCCCTGAGAACAC		1060619-1060636

Lower case text, no italics indicate the GC clamp. Lower case, underlined italics denote restriction sites. T_A=annealing temperature
