# Supplementary material for: Characterisation of a putative M23-domain containing protein in Mycobacterium tuberculosis
Source: PLoS One. 2021 Nov 16;16(11):e0259181. doi: 10.1371/journal.pone.0259181 (PMC8594824; doi:10.1371/journal.pone.0259181)
Supplement: S6 Table — Direct orthologues occur in the same row. Orthologues were identified in the PHMMER database [31]. (PDF) [file pone.0259181.s009.pdf]

Table S6. Mycobacterial expansion of M23 endopeptidases highlights Rv0950c as the most conserved orthologue in *M. tuberculosis*. Direct orthologues occur in the same row. Orthologues were identified in the PHMMER database [1]

M. leprae	***M. abscessus***	***M. tuberculosis***	***M. marinum***	***M. smegmatis***
ML_0514	1055	Rv0950c	4551	5526
	3197	Rv2891	1818	2518
		Rv3786c	5347	-
			3448	-
				1192
				0247

References

1. Eddy SR. Profile hidden Markov models. Bioinformatics. 1998;14(9):755-63. doi: 10.1093/bioinformatics/14.9.755.
