# Supplementary material for: Characterisation of a putative M23-domain containing protein in Mycobacterium tuberculosis
Source: PLoS One. 2021 Nov 16;16(11):e0259181. doi: 10.1371/journal.pone.0259181 (PMC8594824; doi:10.1371/journal.pone.0259181)
Supplement: S7 Table — Data collected from PHMMER, pFAM and Mycobrowser [28,30,31]. (PDF) [file pone.0259181.s010.pdf]

Table S7. Distribution of Rv0950c orthologues in mycobacterial species. Data collected from PHMMER, pFAM and Mycobrowser [1-3]

Group	Species	Locus	Domain architecture
MTB complex	*M. tuberculosis* H37Rv	Rv0950c	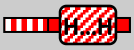
	M. tuberculosis H37Ra	MRa_0957	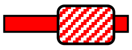
	M. tuberculosis CDC 1551	MT0977	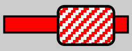
	M. tuberculosis KZN 4207	TBSG_03059	
	M. africanum	RN09_1159	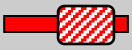
	M. bovis AF122/97	MB_0975c	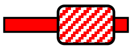
	M. bovis BCG Pasteur	BCG_1004c	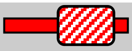
Other pathogens	*M. abscessus* ATCC19977	MAB_1055c	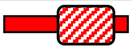
	M. avium	MAV_1073	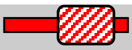
	M. avium subsp. paratuberculosis	MAP_0895c	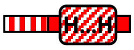
	M. ulcerans AGY99	MUL_4721	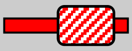
	M. marinum	MMAR_4551	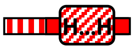
	M. intracellularae	OCU_09440	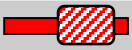
	M. leprae TN	ML0154c	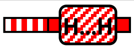
Environmental species	*M. smegmatis*	MSMEG_5526	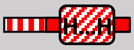
	M. vanbaaleni PYR-1	Mvan_4871	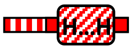
	M. sp KMS	Mkms_4414	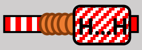
	M. sp JLS	Mjls_4708; 4510	
	M. sp MCS	Mmcs_4328	
	M. gilvum PYR-GCK	Mflv_1864	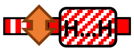
		Mflv_2817	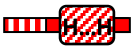

Key	
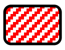	m23 domain
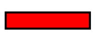	disorder
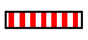	low complexity
**H...H**	catalytic residues
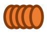	coiled-coil domain
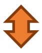	transmembrane domain

References

1. Eddy SR. Profile hidden Markov models. Bioinformatics. 1998;14(9):755-63. doi: 10.1093/bioinformatics/14.9.755.
2. Finn RD, Bateman A, Clements J, Coggill P, Eberhardt RY, Eddy SR, et al. Pfam: The protein families database. Nucleic Acids Research. 2014;42(D1):D222-30. doi: 10.1093/nar/gkt1223.
3. Kapopoulou A, Lew JM, Cole ST. The MycoBrowser portal: A comprehensive and manually annotated resource for mycobacterial genomes. Tuberculosis. 2011;91(1):8-13. doi: 10.1016/j.tube.2010.09.006.
