# Supplementary material for: Characterisation of a putative M23-domain containing protein in Mycobacterium tuberculosis
Source: PLoS One. 2021 Nov 16;16(11):e0259181. doi: 10.1371/journal.pone.0259181 (PMC8594824; doi:10.1371/journal.pone.0259181)
Supplement: S8 Table — (PDF) [file pone.0259181.s011.pdf]

Table S8. Broth microdilution determination of MIC (µg/ml) of cell-wall targeting antibiotics, lysozyme and ROS-generating compounds*.

Compound	RvS	ΔRv0950c	ΔRv0950c::Rv0950c
**Cefamandole**	7.8	7.8	*nd*[ϕ]
**Cefotaxime**	0.5	0.5	*nd*[ϕ]
**Ceftriaxone**	3.9	3.9	*nd*[ϕ]
**Cycloserine**	12.5	12.5	nd
**Meropenem**	0.25	0.5 – 1	0.5 – 1
**Isoniazid**	0.015	0.015	0.015 – 0.03
**Lysozyme**	160	160	160 – 320
**Menadione**	7.8 – 15.6	7.8 – 15.6	7.8 – 15.6

* Data are representative of three independent biological repeats.

ϕ *nd* – not determined
