# Supplementary material for: Characterisation of a putative M23-domain containing protein in Mycobacterium tuberculosis
Source: PLoS One. 2021 Nov 16;16(11):e0259181. doi: 10.1371/journal.pone.0259181 (PMC8594824; doi:10.1371/journal.pone.0259181)
Supplement: S1 Raw images — (PDF) [file pone.0259181.s012.pdf]

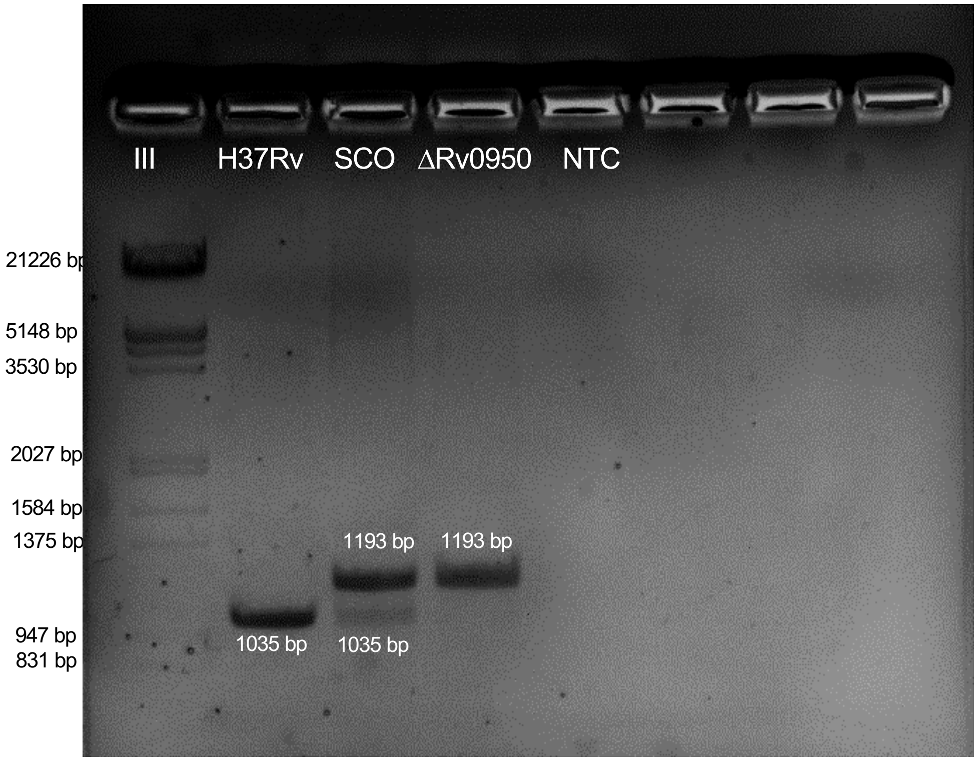

NTC: No Template Control

Agarose gel for PCR confirmation shown in Supplementary Figure S1. Shown is a 2 % agarose gel of PCR products. The gel was cropped and represented in Supplementary Figure S1.

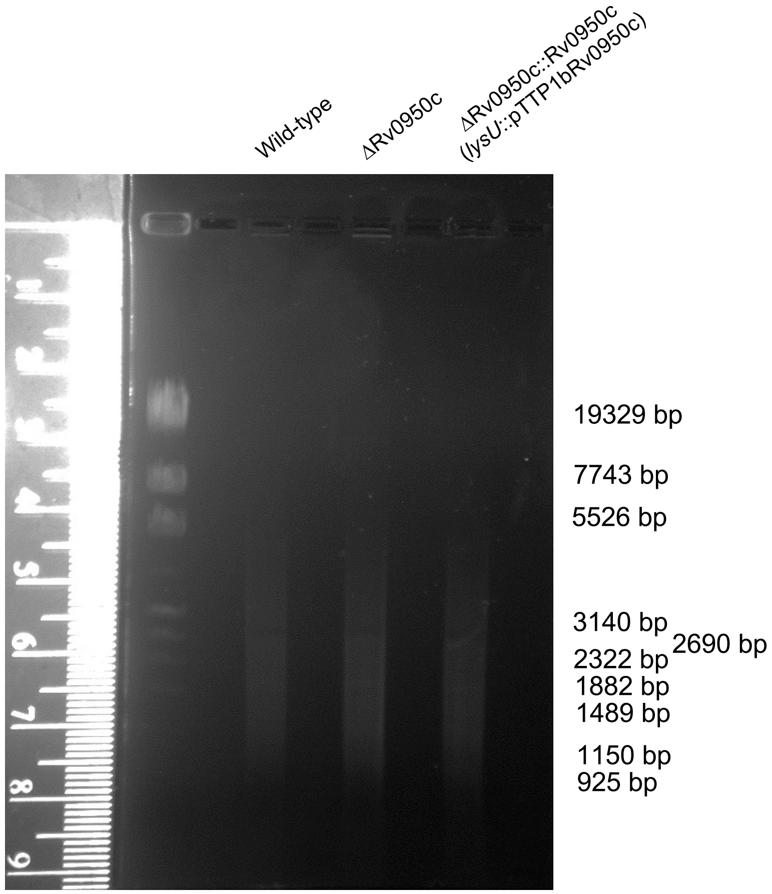

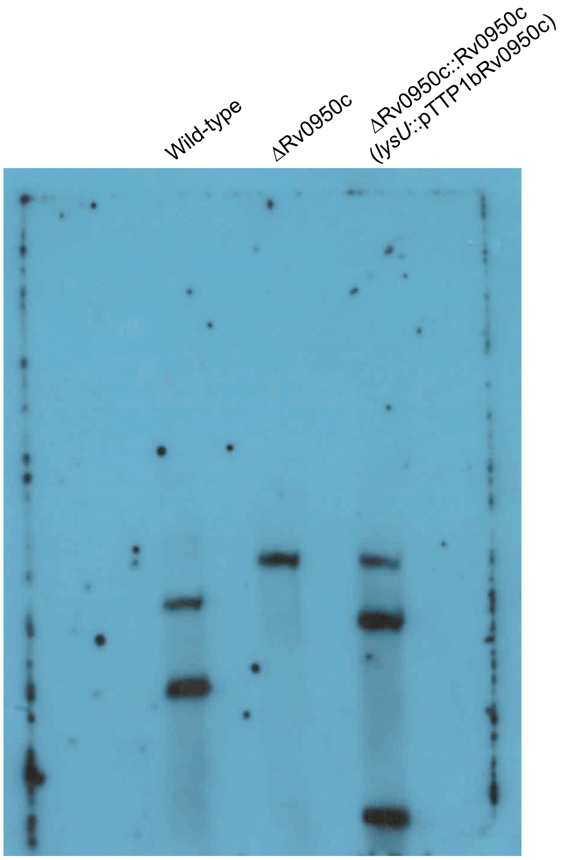

Uncropped *Bgl*I digests of genomic DNA for Southern Hybridizations. The Southern Blot is shown in Figure S2.
